# Hemolytic crisis in a patient treated with eculizumab for paroxysmal nocturnal hemoglobinuria possibly triggered by SARS-CoV-2 (COVID-19): a case report

**DOI:** 10.1007/s00277-020-04318-6

**Published:** 2020-11-10

**Authors:** Hannah Schüller, Franziska Klein, Michael Lübbert, Eric Peter Prager

**Affiliations:** 1 Department of Nephrology, University Medical Center, Faculty of Medicine, University of Freiburg, Freiburg, Germany; 2 Department of Hematology, Oncology & Stem Cell Transplantation, Faculty of Medicine, University of Freiburg, Freiburg, Germany

Dear Editor,

COVID-19 caused by the recently discovered zoonotic severe acute respiratory syndrome coronavirus-2 (SARS-CoV-2) is characterized by high infectiousness and mild to severe respiratory symptoms, to some extent with life-threatening respiratory distress accompanied by multi-organ failure [1–3].

A 68-year-old Caucasian female patient was admitted to our hospital with confirmed COVID-19.

She had been diagnosed with paroxysmal nocturnal hemoglobinuria (PNH) in 1977. Other than that, she has a past medical history significant for hypertension and chronic hepatitis C. For PNH, the patient was started on eculizumab (900 mg/14 days) in 2007.

On March 19, 2020, 13 days after receiving her regular treatment with eculizumab, she presented with subfebrile temperatures, mild cough, a sore throat, and diarrhea. Because of her malaise, her eculizumab dose on March 20 was postponed. On March 21, 2020, she received the positive result for SARS-CoV-2 of the nasopharyngeal swab taken 2 days earlier. Five days later, a drop of hemoglobin levels was recognized, and the patient was admitted to our hospital for suspected recurrent hemolytic crisis.

Vital signs on admission were within normal range. Physical examination showed no abnormalities.

Laboratory parameters on admission on March 26 were suggestive for hemolytic anemia and mild inflammation (hemoglobin 6.5 g/dl (last documented 10.6 g/dl on March 20)), serum LDH 457 U/l, haptoglobin 63 mg/dl (ref. val. 30–200 mg/dl), reticulocytes 276,000/μl (ref. val. 19,800–80,700/μl), free hemoglobin 5.6 mg/dl (ref. < 5 mg/dl), total bilirubin 2.7 mg/dl, direct bilirubin 1.0 mg/dl, CRP 25 mg/l (normal range < 3 mg/l)). Whether her normal haptoglobin level and the only slightly elevated free hemoglobin level represents extravascular hemolysis or an already stabilized intravascular hemolysis situation at the time of admission remained unclear.

Within several days of admission, she developed thrombocytopenia with a minimum of 102,000/μl, neutropenia with a minimum of 420/μl neutrophils, and lymphopenia with a minimum of 800/μl lymphocytes.

Despite laboratory findings of beginning pancytopenia, she did not show any other clinical signs typical of PNH.

We continued treatment with eculizumab on day 7 (March 26) and 20 (April 4) after positive SARS-CoV-2 testing at the usual dose. Additionally, the patient received two red cell concentrates and 2 × 30 m units of G-CSF. We decided against an extra dose of eculizumab because of her relatively stable hemoglobin levels after admission.

The patient’s mild COVID-19 symptoms were treated with supportive anti-inflammatory agents.

The patient stayed for in-patient treatment for a total of 15 days due to local discharge criteria for COVID-19 cases. The hemoglobin level was stable with 11.8 g/dl at discharge.

Two weeks after discharge, the patient received eculizumab as an outpatient. Four weeks after discharge, her hemoglobin level was 12.4 g/dl, LDH was 238 U/l, and haptoglobin was 86 mg/dl, representing clinical remission of her PNH.

A very recent case series of four PNH patients diagnosed with COVID-19 showed that two of those under regular treatment with C5 inhibitors not only showed fewer signs of hemolysis but also milder symptoms of COVID-19, suggesting a reduction of the inflammatory lung damage [4].

Another case series of four patients with confirmed diagnosis of COVID-19 and severe pneumonia showed that eculizumab induced a drop in inflammatory markers [5].

Our case is in line with these observations; interestingly, our patient showed exacerbation of her chronic anemia, suggesting that COVID-19 might trigger hemolytic crises in these patients, possibly in our case this episode was extravascular. We cannot exclude the possibility that the hemolytic episode was facilitated by the postponement of the treatment due to her ill status. This supports that treatment intervals need to be adhered to also in patients proven or suspicious of being SARS-CoV-2-positive.

Immune damage and cytokines released by inflammatory storms in the early stage of COVID-19 might explain why eculizumab as an inhibitor of the complement cascade may be important in SARS-CoV-2 containment [6, 7].

Based on our report and recently published data indicating that PNH patients may present mild symptoms of COVID-19 despite or even because of eculizumab treatment, further studies are needed to validate this observation.
